# Improvement of Lutein and Zeaxanthin Production in *Mychonastes* sp. 247 by Optimizing Light Intensity and Culture Salinity Conditions

**DOI:** 10.4014/jmb.2211.11006

**Published:** 2022-11-30

**Authors:** Seong-Joo Hong, Kyung June Yim, Young-Jin Ryu, Choul-Gyun Lee, Hyun-Jin Jang, Ji Young Jung, Z-Hun Kim

**Affiliations:** 1 Department of Biological Engineering, Inha University, Incheon 22212, Republic of Korea; 2 Industry-Academia Interactive R&E Center for Bioprocess Innovation, Inha University, Incheon 22212, Republic of Korea; 3 Microbial Research Department, Nakdonggang National Institute of Biological Resources, Sangju 37242, Republic of Korea; 4 Laboratory of Chemical Biology and Genomics, Korea Research Institute of Bioscience and Biotechnology, Daejeon 34141, Republic of Korea

**Keywords:** Microalgae, *Mychonastes* sp., lutein, zeaxanthin, central composite design

## Abstract

In this study, we sought to improve lutein and zeaxanthin production in *Mychonastes* sp. 247 and investigated the effect of environmental factors on lutein and zeaxanthin productivity in *Mychonastes* sp. The basic medium selection and N:P ratio were adjusted to maximize cell growth in one-stage culture, and lutein and zeaxanthin production conditions were optimized using a central composite design for two-stage culture. The maximum lutein production was observed at a light intensity of 60 μE/m^2^/s and salinity of 0.49%, and the maximum zeaxanthin production was observed at a light intensity of 532 μE/m^2^/s and salinity of 0.78%. Lutein and zeaxanthin production in the optimized medium increased by up to 2 and 2.6 folds, respectively, compared to that in the basic medium. Based on these results, we concluded that the optimal conditions for lutein and zeaxanthin production are different and that optimization of light intensity and culture salinity conditions may help increase carotenoid production. This study presents a useful and potential strategy for optimizing microalgal culture conditions to improve the productivity of lutein and zeaxanthin, which has applications in the functional food field.

## Introduction 

Microalgae are photosynthetic organisms that produce various pigments that absorb sunlight over a wide range of wavelengths. Excessive sunlight and environmental stress can result in the production of free radicals in cells, which attack and damage cells, while the pigments present in microalgae specialize in defense mechanisms. As pigments, carotenoids play a vital role in photosynthesis and provide energy to cells by absorbing light and dissipating energy to protect the photosynthetic apparatus [1, 2]. Carotenoids are divided into primary and secondary carotenoids, and are produced from their precursor, lycopene. Primary carotenoids include the a-carotene-derived lutein and loroxanthin, which act as accessory pigments for transferring absorbed energy to the chlorophyll [3]. Secondary carotenoids are produced from β-carotene and synthesized in various forms, including zeaxanthin, canthaxanthin, and astaxanthin. Under stressful conditions, cells form a protective layer to prevent damage to the photosynthetic apparatus [4].

The antioxidant properties of carotenoids can mitigate the harmful effects of free radicals and hence can protect humans from various diseases, such as early aging, specific cancers, and damaged immune responses [1, 5]. Lutein and zeaxanthin are present in the retina and lens of the eyes and are known to protect the eyes via their antioxidant action. Therefore, proper intake of these products prevents or improves degenerative diseases such as age-related macular degeneration (AMD) and cataracts [6, 7]. Nowadays, the use of eye health functional foods, such as lutein and zeaxanthin, is attracting increasing interest as a practical means to delay cataracts and phagocytosis. Indeed, the number of people consuming lutein-rich foods, beverages, and health-functional foods is increasing [8]. Meanwhile, microalgae, which are a source of carotenoids, are gaining popularity because of consumer preferences for natural products. Despite its many advantages, carotenoid production from microalgae is currently experiencing a bottleneck due to challenges arising from the growth medium, the high cost of the cultivation process, extraction, and commercial-scale microalgae biological processes [9].

In the past, attempts have been made to enhance carotenoid production by manipulating nutritional and environmental factors and related genes [10-13]. The production of lutein, which is a growth-coupled metabolite, is influenced by culture conditions such as temperature, light intensity, photoperiod, carbon dioxide concentration, nutrients, and salinity [14-17]. Furthermore, the intracellular content of zeaxanthin increases under extreme stress conditions. Thus, strong light, nitrogen, phosphate depletion, and salt addition are effective strategies for increasing zeaxanthin content [14, 18, 19]. Manipulation of light intensity may alter the synthesis mechanism of lutein and zeaxanthin influencing the photosynthetic system of microalgae [20]. When the salinity of the culture is high, the resulting osmotic stress and ion imbalance cause free radical production and induce carotenoid synthesis [21].

Previously, *Mychonastes* was mainly studied as a lipid producer for biodiesel or nervonic acid production; however, studies on growth potential and pigment production are yet to be reported [22-24]. In this study, we aimed to determine the optimal conditions for increasing lutein and zeaxanthin production in the newly isolated microalgal strain *Mychonastes* sp. 247 by controlling light intensity and culture salinity. To this end, we selected the basic medium for the first-stage culture and investigated the cell composition of *Mychonastes* sp. 247 to determine the N/P ratio adjustment in the medium. Furthermore, the environmental conditions for *Mychonastes* sp. 247 were optimized to improve lutein and zeaxanthin production by applying a central composite design (CCD).

## Materials and Methods

### Strain and Culture Conditions

As previously reported, the green microalga *Mychonastes* sp. 247 was locally isolated from a weir on the freshwater Jangdongjae Reservoir (Korea) [25]. The cells were cultivated in a 250 ml Erlenmeyer flask, containing 100 ml fresh BG-11 medium (Sigma-Aldrich, USA), at 22 ± 2°C under continuous illumination from fluorescent lamps at a constant intensity of 100 μE/m^2^/s, with shaking at 120 ×*g*.

To optimize the appropriate culture medium for *Mychonastes* sp., the cultures were grown in BG-11 and Bold's Basal Medium (BBM; Sigma-Aldrich). Details of the culture media are presented in Table 1. *Mychonastes* sp. was cultivated in a 0.5 L bubble column photobioreactor containing 400 ml BG-11 or BBM under continuous fluorescent lamp illumination at a constant intensity of 100 μE/m^2^/s, with aeration by 2% CO_2_ balanced with air at 0.1 vvm.

### Biomass Determination and Composition Analysis

Algal culture samples were collected every 24 h. The optical density of the *Mychonastes* sp. culture was measured spectrophotometrically at 680 nm. Briefly, the OD680, obtained using a spectrophotometer (Perkin Elmer, LAMBDA 465, USA), was converted to dry cell weight (DCW) concentration (g/l) based on a linear relationship between the OD680 and DCW, which was obtained after extensive data analysis and is given by Eq. 1 for *Mychonastes* sp. as follows [26]:

$$\text{Dry cell weight (g/l) = 0.1537 }\times {\text{OD680 (R}}^{\text{2 }}\text{= 0.99).}$$ (1)

The blank used was BG-11 medium before incubation, and all samples were measured thrice. The C, H, N, and S intracellular components were analyzed using an elemental analyzer (Thermo EA1112, Thermo Fisher Scientific, USA), and P was measured using an inductively coupled plasma spectrophotometer (ICP-OES, OPTIMA 7300DV; Perkin Elmer, USA).

### Pigment Analysis

The harvested cells were centrifuged at 2,000 ×*g* for 20 min and washed twice with deionized water. Prior to carotenoid analysis, the cells were freeze-dried using a freeze dryer (FD8512; Ilshinbiobase, Korea). Dry cells (200 mg) were mixed with an acetone-water mixture (10 ml, 90% v/v acetone) and sonicated using a sonicator (Powersonic 410, Hwashin Tech, Korea) for 7 min. Carotenoid analyses were performed using high-performance liquid chromatography (HPLC; Agilent, USA) and a reversed-phase Luna 3 μm C8(2) 100 Å 150 × 4.6 mm analytical column (Phenomenex, USA). The mobile phase consisted of a mixture of methanol-water (7:3, v/v) with 100% methanol (A) and 22 mM ammonium acetate with acetate buffer (B). The gradient started with 5% A-35% A (15 min), 35% A (5 min), 35% A-60% A (15 min), 60% A-100% A (10 min), and 100% A (10 min). The flow rate was 1 ml/min, and the chromatogram was monitored at 450 nm. Lutein, zeaxanthin, β-carotene, and chlorophyll were identified using authentic standards (Sigma-Aldrich).

### Central Composite Design (CCD)

The culture for the CCD experiments was performed in a bubble column photobioreactor (BC-PBR) with a working volume of 400 ml. The basic medium was modified BG-11, and as shown in Table 2, the salt concentration of each experimental group was set differently according to the level of the experimental factor. During the culture period, air containing 2% CO_2_ was supplied, and light energy was continuously irradiated. The light intensity applied to BC-PCR was adjusted according to the conditions of each laboratory, as shown in Table 2. The incubation period was 10 days; the biomass and pigment production were measured on the 10th day; and the results were analyzed using statistical software. Lutein and zeaxanthin production (g/l) was used as the result factors for each experimental group (Eq. 2):

$$y=\beta_{0}+\sum_{i=1}^{k}\beta_{i}x_{i}+\sum_{i=1}^{k}\beta_{ii}x_{i}^{2}+\sum \sum_{i<j}\beta_{ij}x_{i}x_{j}+\varepsilon$$ (2)

where *y* is the predicted outcome factor, *β0* is the intercept of the plane, *βi* is the coefficient of the equation, *x* and *y* are the coded levels of the variable, and ε is the error term. The results were analyzed using a surface plot and contour plot to optimize cell production and growth rate, and the optimal value was derived using an optimizer of the software Minitab.

### Statistical Analysis

Statistical analysis for CCD was performed using lutein and zeaxanthin as response factors, and Minitab (v.14.12) was used in the analysis design. Regression variables for the response surface secondary model are shown in Tables 3 and 4. The fit quality of the polynomial model was expressed using a correlation coefficient, and the statistical significance was tested by the F-test of the software Minitab. The analysis of variance (ANOVA) tested the suitability of the quadratic polynomial equation for the experimental data.

## Results and Discussion

### Basic Culture Medium Determination

**Fig. 1:**
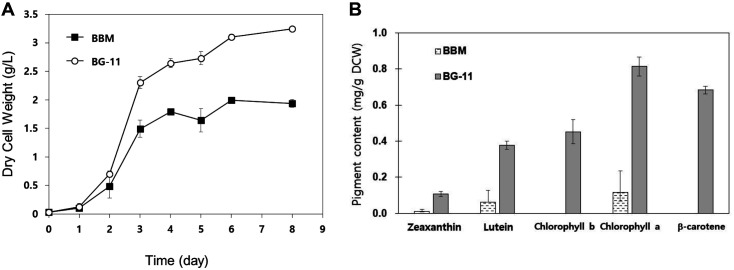
Profiles of (A) dry cell weight (g/l) and (B) pigment content (g/g dry cell weight [DCW]) in various media. *Mychonastes* sp. 247 cultured in BBM (■), and BG-11 (○).

When using BBM, which can be used to cultivate various green algae, soil extraction or vitamins are not required; therefore, it was selected as the medium for culturing *Mychonastes* sp. [27-29]. BBM is advantageous for axenic cultures, since inorganic substances (as the main ingredient) are resistant to contamination, as shown in Table 1. BG-11, an optimized medium suitable for the growth and maintenance of blue-green algae [30], mainly comprises inorganic components and has a high concentration of nitrate, as shown in Table 1. *Mychonastes* sp. were cultured in the BBM and BG-11 media for eight days. As shown in Fig. 1A, the BBM and BG-11 media showed exponential and stationary phases, and cells grew without delay. The logarithmic cell growth graph results indicated that cell growth was normal in the BBM and BG-11 media. The biomass increased to 3.24 g/l in BG-11 medium (the largest biomass increase) and to 1.94 g/l in BBM medium. BG-11 medium exhibited 40% greater biomass than the BBM medium, which confirmed the advantage of BG-11 medium for culturing *Mychonastes* sp. over BBM.

Zeaxanthin, lutein, chlorophyll *a*, chlorophyll *b*, and β-carotene contents were measured to confirm pigment production in *Mychonastes* sp. cells cultured in BG-11 and BBM media (Fig. 1B). Compared with those in *Mychonastes* grown in the BBM, the contents of all pigments were substantially higher in *Mychonastes* grown in the BG-11 medium. In particular, the levels of chlorophylls *a* and *b*, which are associated with the growth potential, were high (Fig. 1B). Chlorophylls *a* and *b* are light-harvesting complexes of photosystems I and II; accordingly, the higher their content, the higher the ability to absorb solar energy. Therefore, the biomass in the BG-11 medium was higher than that in other media. In addition, the levels of zeaxanthin, lutein, and β-carotene, which contain only carbon and hydrogen, were also higher in BG-11 than in BBM. As photosynthesis efficiency increases with an increase in chlorophyll *a* and *b* contents, we predicted that a metabolism pathway other than carbon metabolism of biomass would induce the accumulation of carotenoids such as zeaxanthin, lutein, and β-carotene [31]. Although it is generally known that nutrient deficiency induces the synthesis of carotenoids, a previous study has suggested that this varies depending on the carotenoid type [32]. The BBM is known to be suitable for the growth and maintenance of eukaryotic microalgae [33]. However, a previous study on the production of useful substances confirmed that *Mychonastes* sp. shows maximum growth in BG-11 medium [23, 34]. Therefore, we used BG-11 as the basic medium, which provided the highest cell growth and carotenoid production.

### Analysis of Cell Composition and the N/P Ratio

A cell is composed of C, H, N, P, and S. The N content of microalgae is crucial for the synthesis of chlorophylls *a* and *b*, as well as the production of amino acids. Furthermore, P is a major factor affecting photosynthesis, metabolism, and energy-related compounds [35-38]. Accordingly, we assessed the intracellular cell components to determine the contents of N and P and to adjust the culture medium conditions. *Mychonastes* were analyzed to determine the ratios of C, H, N, S, and P, as shown in Fig. S1A. The proportions of N and P, which are major components affecting the growth of microalgae, were 7% and 1%, respectively. The N:P ratio represents an important nutritional factor for microalgal growth. Accordingly, designing a medium reflecting this ratio is crucial for cell growth; hence, N and P should not be added in excess. BG-11 is a medium modeled on cyanobacteria, and, as mentioned above, the N:P ratio of the medium was 44, indicating an excessive nitrogen concentration. In this case, P concentration limits the growth, whereafter the cells can no longer multiply.

To increase the biomass in BG-11, the concentrations of N and P, as the most important components of the microalgae medium, were calculated in each medium. The tested media used included NaNO_3_ as nitrate source; the BBM contained two forms of phosphate, and the BG-11 contained one form of phosphate. Both N and P, which are present in nitrates and phosphates, are cell components; therefore, their presence in the medium stimulates growth. Cell growth was affected by the amount of N in the medium; the highest growth was observed in BG-11, a medium containing a large amount of N and P. We speculate that the amount of P, which was less than that of N in BG-11, is the limiting factor for cell growth.

**Fig. 2:**
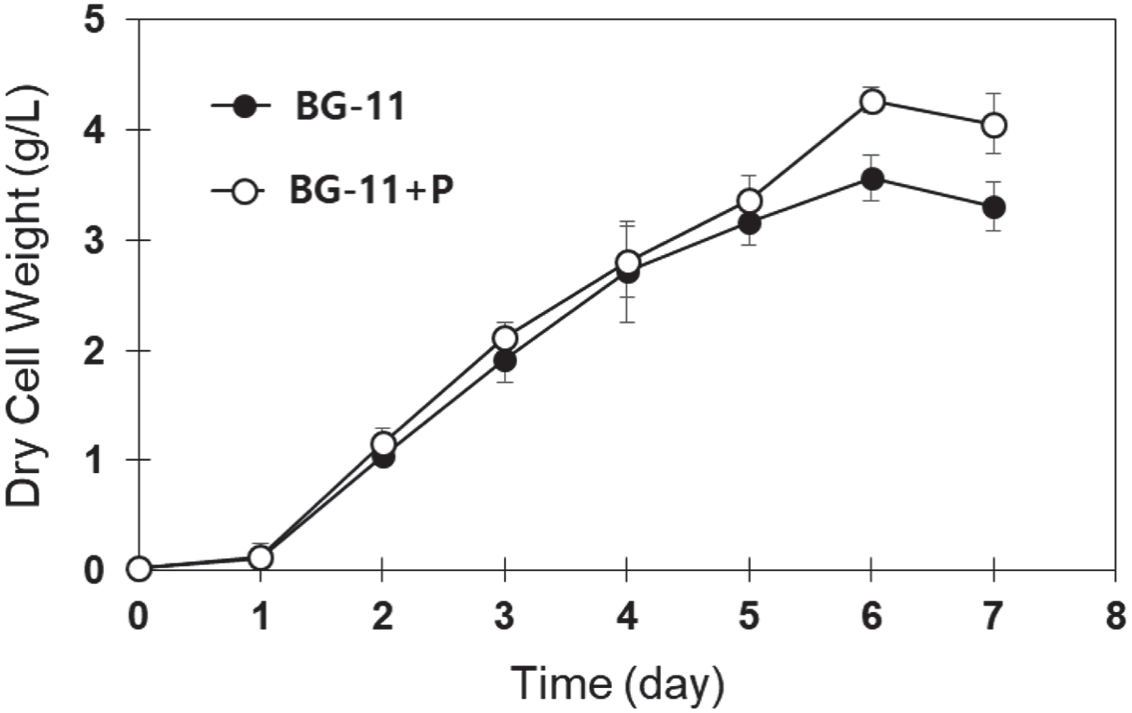
Profiles of dry cell weight (DCW) (g/l) in BG-11 and BG-11 with added phosphate (BG-11+P).

A culture experiment was conducted using BG-11 medium as the basic medium based on the N:P ratio derived from the cell member analysis data. The control group (BG-11) was a BG-11 medium with an N:P ratio of 44, and the experimental group (BG-11+P) was a BG-11 medium with an N:P ratio adjusted to 7 based on the cell composition analysis data. After seven days of incubation, the cells in BG-11 and BG-11+P showed similar growth until day 4; however, the difference in growth from day 5 onwards showed that the experimental group exhibited high cell production in the stationary phase (Fig. 2). As described above, in the case of BG-11, cell growth was limited on day 4 because of the low P concentration compared to the high N concentration; however, the experimental group continued growing until day 6 due to a higher availability of P. This suggests a limitation of cell growth under P deficiency, as this affects chlorophyll content [39]. In addition, photosynthesis, which has a significant impact on the growth and metabolism of microalgae, is suppressed [40, 41]. In our next study, we intend to conduct experiment by altering the N:P ratio to 7 by adding P.

### Selection of Optimal Pigment Production Conditions

To optimize the light and culture salinity conditions affecting lutein and zeaxanthin production, two-stage culture conditions were investigated using a CCD. Biomass and pigment contents were measured after incubation for 10 days according to the CCD experimental plan (Fig. S2). Table 2 shows the variables and conditions used to optimize the culture environment. Thirteen experiments were conducted and statistically analyzed as shown in Tables 3 and 4. The statistical significance of the variables and their interactions at various probability levels were described based on distribution tests and analysis of variance fitted to quadratic polynomial equations. The results revealed that culture salinity influenced lutein production, and both light intensity and salinity had significant effects on zeaxanthin production. No interaction was observed between culture salinity and light intensity during lutein and zeaxanthin production (Eqs. 3, 4). The significance of the CCD models was predicted as the coefficient of determination values, and the R^2^ values represent 85.9% and 85.5% variability in lutein and zeaxanthin production, respectively.

$${\text{Y}}_{\text{lutein}}\text{=1954.71-58.35×Light-758.42×NaCl+113}{\text{.65×Light}}^{\text{2}}\text{-783}{\text{.14×NaCl}}^{\text{2}}\text{-73.82×Light×NaCl}$$ (3)

$${\text{Y}}_{\text{zeaxanthin}}\text{=563.3+168.71×Light-28.63×NaCl-90}{\text{.03×Light}}^{\text{2}}\text{-326}{\text{.43×NaCl}}^{\text{2}}\text{-57.26×Light×NaCl}$$ (4)

Here, Y_lutein_ and Y_zeaxanthin_ represent the predicted lutein and zeaxanthin production, respectively.

**Fig. 3:**
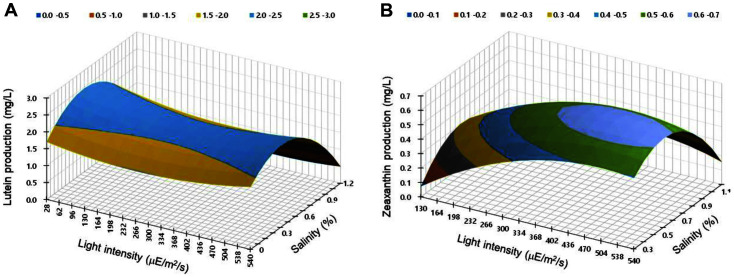
Three-dimensional surface plots of (A) lutein production (mg/l) and (B) zeaxanthin production (mg/l) as functions of light intensity and salinity.

A three-dimensional plot was used to confirm the optimal values for lutein and zeaxanthin production and to visualize the relationship among reaction value, light intensity, and salinity. The effects of light intensity and culture salinity on lutein and zeaxanthin production are shown in Fig. 3. Lutein production increased with reduction in luminosity and culture salinity, and zeaxanthin production increased with an increase in light intensity and salinity.

Lutein is a growth-coupled metabolite and affects biomass; however, lutein content has previously been shown to increase under stress conditions induced by slight changes in salinity [42]. Lutein content in Murielopsis sp. has been shown to increase under moderate saline conditions; additionally, increased content of zeaxanthin was observed compared to that of lutein under high salinity conditions. However, high salinity inhibited cell growth, which in turn negatively affected zeaxanthin production [43]. In our study, the production of zeaxanthin decreased even above optimal saline conditions because of a decrease in the experimental cell biomass.

**Fig. 4:**
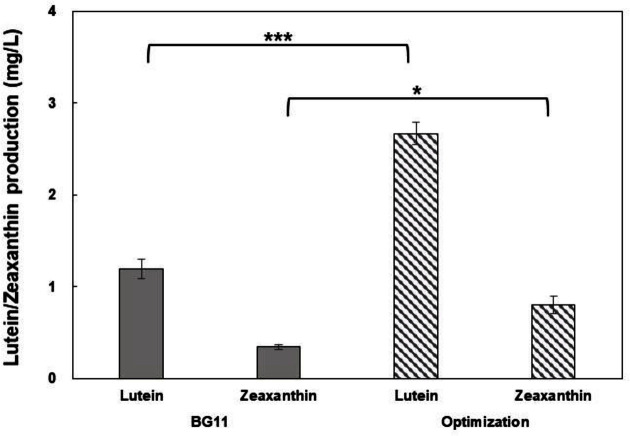
Comparison of lutein and zeaxanthin yield (mg/l) obtained using BG-11 under optimized conditions. Data represent the mean ± SEM from triplicates. *p < 0.05, ***p < 0.001 versus lutein and zeaxanthin from BG-11 (control).

Light intensity is an important factor limiting biomass and carotenoid production. The *Mychonastes* sp. used in this study showed optimal lutein production at a low light intensity and optimal zeaxanthin production at high light intensity. Microalgae also show increased lutein content, structurally coupled to the photosynthetic apparatus, due to the increased effectiveness of the photo-adaptive effect to improve photosynthetic efficiency at a low light intensity [44, 45]. In contrast, zeaxanthin is synthesized to protect the photosynthetic apparatus from excess light energy and is thus produced in high concentrations at high light intensities [46]. The highest lutein content was observed in *Cocomyxa onubensis* at 50 μE/m^2^/s, and Tetraselmis sp. CTP4 achieved higher lutein content at 170 μE/m^2^/s compared to 33 μE/m^2^/s [47, 48]. In addition, the transcriptomic study of Desmodesmus sp. JSC3 showed that the lutein synthesis genes of β-carotene hydroxylase and ε-carotene hydroxylase were upregulated under low illumination and initial nitrogen deficiency conditions [49]. This has been explained as a reason why high concentrations of lutein are essential for maintaining the structure of light harvest complex and enhancing photosynthesis efficiency under low-light conditions [43]. Conversely, *Scenedesmus almeriensis* and *Parachlorella* sp. JD-076 showed high lutein content at 1000 μE/m^2^/s or higher, showing behavior of lutein accumulation at high light intensity [42, 50]. Therefore, since the light conditions for the accumulation of lutein exhibit strain-specific characteristics, it is necessary to test and optimize light conditions to improve lutein productivity. Lutein production was maximum (2.58 mg/l) under the following conditions: light intensity = 60 μE/m^2^/s and salinity = 0.49%; zeaxanthin production was maximum (0.87 mg/l) at a light intensity of 532 μE/m^2^/s and salinity of 0.78%. In addition, lutein and zeaxanthin production increased by 2 and 2.6 times, respectively, compared to those in the unoptimized BG-11 medium (Fig. 4).

This study identified media that are effective at increasing lutein and zeaxanthin production under complex stress conditions of salinity and light intensity. In addition, we optimized the production of lutein and zeaxanthin with different synthetic mechanisms, by adjusting culture salinity and light intensity. We demonstrated that *Mychonastes* sp. 247 strain is an ideal microalga that can selectively produce lutein and zeaxanthin through environmental control.

In this study, we formulated a medium suitable for enhancing biomass in the native microalga *Mychonastes* sp. 247, by adjusting the N:P ratio. A CCD was used to maximize lutein and zeaxanthin production in this strain by controlling salinity and light conditions, respectively. Culture optimization yielded 2- and 2.6-fold increases in lutein and zeaxanthin production, respectively, compared to that in conventional media. These findings indicate that lutein and zeaxanthin can be produced from *Mychonastes* sp. by manipulating light intensity and culture salinity conditions.

## Supplemental Materials

Supplementary data for this paper are available on-line only at http://jmb.or.kr.

## Figures and Tables

**Table 1:** Comparison of the contents of nutrients present in the BBM, and BG-11 used for culturing.

Component	BBM (mM)	BG-11 (mM)
NaNO_3_	2.94	17.65
K_2_HPO_4_	0.43	0.18
KH_2_PO_4_	1.29	0.00
NaH_2_PO_4_ · H_2_O	0.00	
MgSO_4_ · 7H_2_O	0.30	0.30
CaCl_2_ · 2H_2_O	0.17	0.24
NaCl	0.43	0.00
Na_2_EDTA	0.15	0.00
KOH	0.55	0.00
FeSO_4_ · 7H_2_O	0.02	0.00
Citric acid	-	0.03
Ferric ammonium citrate	-	0.02
Na_2_CO_3_	-	0.19

Trace elements	(µM)	(µM)

H_3_BO_3_	184.36	46.25
ZnSO_4_ · 7H_2_O	15.37	0.77
MnCl_2_ · 4H_2_O	72.76	9.15
MoO_3_	4.93	-
CuSO_4_ · 5H_2_O	2.28	0.32
Co(NO_3_)_2_ · 6H_2_O	1.68	170.08
NaMoO_4_ · 2H_2_O	-	1.78

**Table 2:** Coded levels and real values of variables in the central composite design.

Run Order	Light intensity (μE/m^2^/s)			Salinity (%)	
	
	Coded	Real		Coded	Real
1	0	300		0	0.7
2	-1.41	60		0	0.7
3	0	300		0	0.7
4	0	300		0	0.7
5	1.41	540		0	0.7
6	-1	130		-1	0.2
7	0	300		0	0.7
8	0	300		0	0.7
9	1	470		-1	0.2
10	0	300		-1.41	0
11	1	470		1	1.2
12	0	300		1.41	1.4
13	-1	130		1	1.2

**Table 3:** Statistical analysis of the coefficients for lutein production.

Variables	Coefficient	*t*-test	*p*-value
Constant	1954.71	9.386	-
Light	-58.35	-0.354	27%
NaCl	-758.42	-4.606	**
Light*Light	113.65	0.644	46%
NaCl*NaCl	-783.14	-4.435	**
Light*NaCl	-73.82	-0.317	34%

*p*-levels of coefficients are given as **99%, *90% by *t*-test.

**Table 4:** Statistical analysis of the coefficients for zeaxanthin production.

Variables	Coefficient	*t*-test	*p*-value
Constant	563.3	6.428	-
Light	168.71	3.061	**
NaCl	-28.63	-0.52	38%
Light*Light	-90.03	-1.523	83%
NaCl*NaCl	-326.43	-5.523	**
Light*NaCl	-57.26	-0.735	51%

*p*-levels of coefficients are given as **99%, *90% by *t*-test.
