# Supplementary material for: Improvement of Lutein and Zeaxanthin Production in Mychonastes sp. 247 by Optimizing Light Intensity and Culture Salinity Conditions
Source: J Microbiol Biotechnol. 2022 Nov 30;33(2):260–7. doi: 10.4014/jmb.2211.11006 (PMC9998206; doi:10.4014/jmb.2211.11006)
Supplement: Supplementary file 1 [file jmb-33-2-260-supple.pdf]

J. Microbiol. Biotechnol.
https://doi.org/10.4014/jmb.2211.11006

Supplementary Figures

Improvement of lutein and zeaxanthin production in *Mychonastes* sp. 247 by optimizing light intensity and culture salinity conditions

.

Seong-Joo Hong[1,2†], Kyung June Yim[3†], Young-Jin Ryu[1], Choul-Gyun Lee[1,2], Ji Young Jung[3], and Z-Hun Kim[3*]

1. Department of Biological Engineering, Inha University, Incheon 22212, Republic of Korea

2. Industry-Academia Interactive R&E Center for Bioprocess Innovation, Inha University, Incheon 22212, Korea

3. Microbial Research Department, Nakdonggang National Institute of Biological Resources, Sangju 37242, Republic of Korea

[†]These authors contributed equally to this work.

*Corresponding author: Z-Hun Kim

E-mail: kimzhun@nnibr.re.kr

(A)

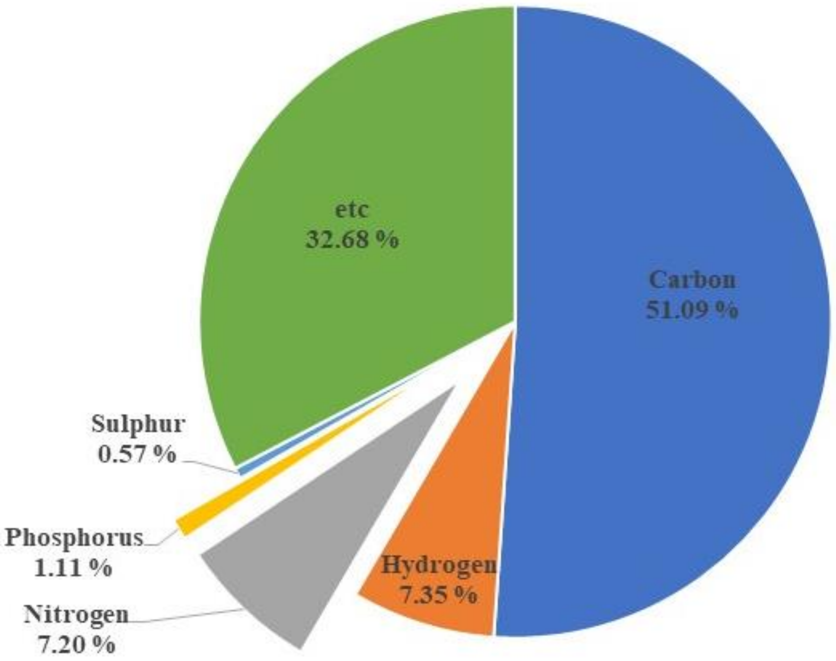

(B)

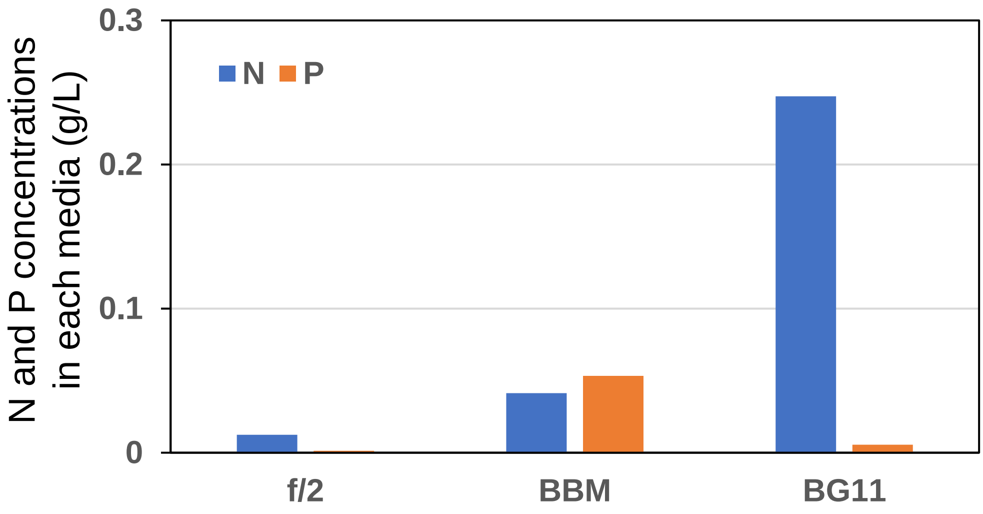

Fig. S1. (A) Proportion of intracellular components of *Mychonastes* sp.; (B) comparison of N and P concentrations in BBM and BG-11.

(A)

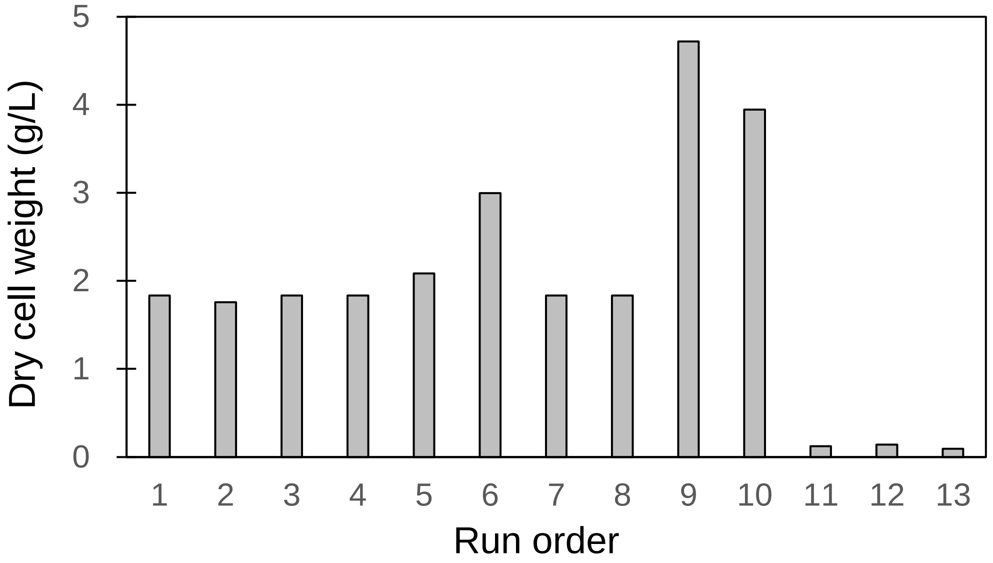

Dry cell weight (g/L)
0
1
2
3
4
5
1 2 3 4 5 6 7 8 9 10 11 12 13
Run order

(B)

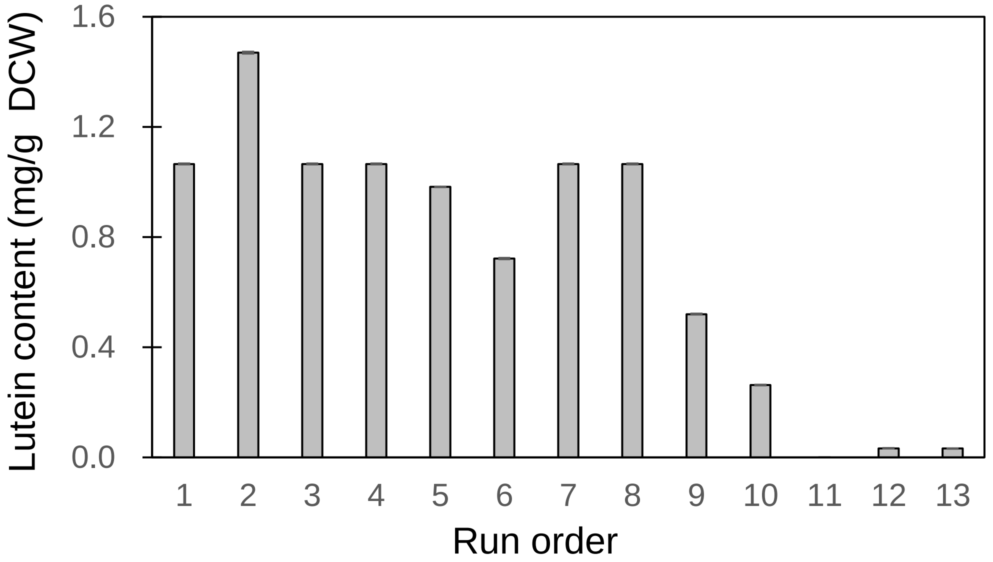

Lutein content (mg/g DCW)
0.0
0.4
0.8
1.2
1.6
1 2 3 4 5 6 7 8 9 10 11 12 13
Run order

(**C**)

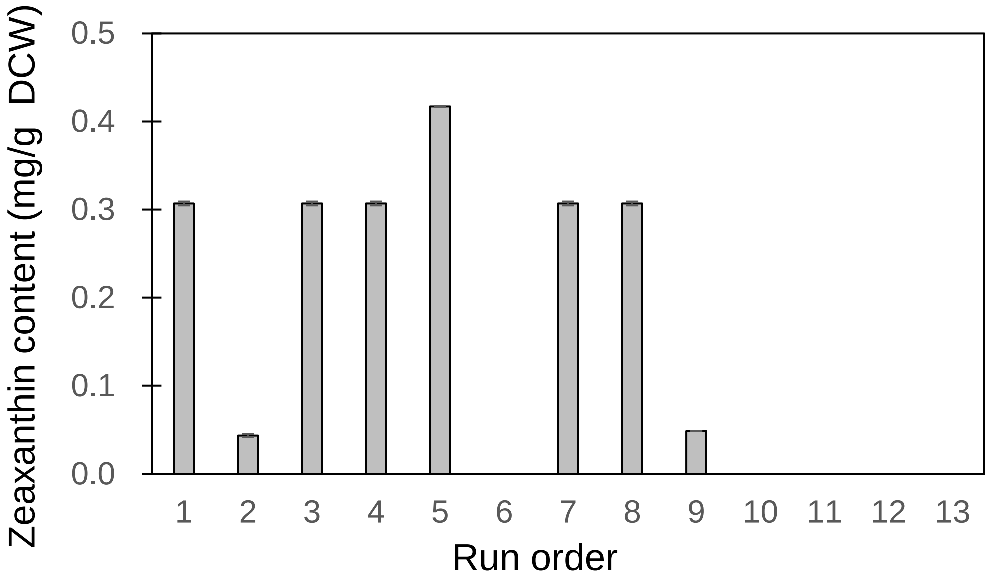

Fig. S2. Profiles of (**A**) dry cell weight (g/L), (**B**) zeaxanthin content (mg/g dry cell weight [DCW]), and (**C**) lutein content (mg/g DCW) in the central composite design (CCD).
